# Variation in blood pressure and heart rate of radiological technologists in worktime tracked by a wearable device: A preliminary study

**DOI:** 10.1371/journal.pone.0276483

**Published:** 2022-11-17

**Authors:** Ryogo Minamimoto, Yui Yamada, Yasuharu Sugawara, Megumi Fujii, Kazuki Kotabe, Kakeru Iso, Hiroki Yokoyama, Keiichi Kurihara, Tsubasa Iwasaki, Daisuke Horikawa, Kaori Saito, Hironori Kajiwara, Futoshi Matsunaga

**Affiliations:** Department of Radiology, Department of Radiology, National Center for Global Health and Medicine, Tokyo, Japan; University of Siena: Universita degli Studi di Siena, ITALY

## Abstract

The aim of this preliminary study was to measure the systolic BP (SBP) and diastolic BP (DBP) and heart rate (HR) of radiological technologists by WD, and evaluate variation among individuals by worktime, day of the week, job, and workplace. Measurements were obtained using a wristwatch-type WD with optical measurement technology that can measure SBP and DBP every 10 minutes and HR every 30 minutes. SBP, DBP, and HR data obtained at baseline and during work time were combined with the hours of work, day of the week, job, and workplace recorded by the participants in 8 consecutive weeks. We calculated the mean, the ratio to baseline and coefficient of variation [CV(%)] for SBP, DBP, and HR. SBP, DBP, and HR values were significantly higher during work hours than at baseline (p<0.03). The ratio to baseline values ranged from 1.02 to 1.26 for SBP and from 1.07 to 1.30 for DBP. The ratio to baseline for SBP and DBP showed CV(%) of approximately 10% according to the day of the week and over the study period. For HR, ratio to baseline ranged from 0.95 to 1.29. The ratio of mean BP to baseline was >1.2 at the time of starting work, middle and after lunch, and at 14:00. The ratio to baseline of SBP were 1.2 or more for irradiation, equipment accuracy control, registration of patient data, dose verification and conference time, and were also working in CT examination room, treatment planning room, linac room, and the office. CV(%) of BP and HR were generally stable for all workplaces. WD measurements of SBP, DBP, and HR were higher during working hours than at baseline and varied by the individuals, work time, job, and workplace. This method may enable evaluation of unconscious workload in individuals.

## Introduction

Hypertension is a major risk factor for death and future disability [1]. Reduced heart rate (HR) variability can be caused by poor autonomic nervous system adaptability, and it is associated with fatigue, stress, and overtraining that can be an early indication of illness and infection [2–4]. Although hypertension is rare in young people, most are aware of their normal blood pressure (BP) values, which are monitored at health checks performed once or twice annually or by self-monitoring at home. Traditional clinical research aiming at monitoring of patients has also evaluated BP at a single time point or longitudinally at select time points. BP and HR are generally measured in the sitting position and at rest, which is a different situation to when working during the day, which takes up at least one third of the day. Therefore, insufficient data have been captured to enable evaluation of the influence of variation in BP and HR during working hours on workers’ health.

Wearable device (WD) technologies can track large amounts of real-time activity and physiological data and convert it to digital data. Several clinical studies have shown that WD data can be collected effectively at scale [5–7]. A comparison of wrist-type and arm-type BP monitoring found fair agreement between the two methods for the ambulatory condition [8].

Several studies have investigated the usefulness of WD for monitoring BP and HR in patients [9–11]. Environmental factors, physical activity, and psychological stress all influence BP and HR. With the aim of increasing patient safety in the operating room, WD has also been applied to continuous BP monitoring to detect sudden changes in BP [12]. Although the accuracy of WD for HR during physical activity was presented in some studies, there has not been enough information on the accuracy it for BP.

There is a known positive association between high levels of stress at work and elevated systolic BP (SBP), and to a lesser extent with diastolic BP (DBP) [13–20]. We hypothesized that variation in BP and HR while at work could be an indicator of overwork and stressful work circumstances, and that such data could be used to improve the working environment and thus maintain the health of workers. It is uncertain whether overwork and work-related stress occur in young radiological technologists, who perform various types of work, including scanning, radiotherapy, equipment checks, and patient care. In this preliminary study, we measured SBP, DBP, and HR by WD for 8 consecutive weeks in young radiological technologists, and evaluated variation in these values between individuals and according to hour of work, day of the week, job, and workplace.

## Materials and methods

### Participants

This prospective study was approved by the National Center for Global Health and Medicine institutional review board (Approved number: NCGM-G-004050-00), and written informed consent was obtained from all participants prior to the study. Included in the study were seven radiological technologists working at our hospital (six men, one woman; mean age (±SD), 29.6±4.0 years; age range, 22–35 years). Mean duration of total work experience was 7.2±3.1 years and mean duration of work experience in the division was 3.6±3.6 years. Four of the seven technologists worked in the division of radiation therapy (mean experience in that division, 8.5 years) and the remaining three worked in the division of nuclear medicine (mean experience in that division, 5.5 years). No participant had any history of illness or was taking medication during the study period. Participants’ work descriptions and work locations are listed in Table 1.

**Table 1:** List of job and workplace.

Job	Workplace
Conference ^†^	CT examination room
CT scan for therapeutic planning *	Linac room
Data analysis ^†^	Lunchroom
Dealing with patients *	Office
Dose verification*	PET examination room
Equipment accuracy control *	Preparation room for radiopharmaceutical
Equipment check*	Scintigraphy room
Irradiation*	Treatment planning room
Preparation of radiopharmaceutical *	
Radiotherapy planning ^†^	
Registration of patient data ^†^	
Research *	
Rest ^†^	
Scanning ^†^	

* standing job,

^†^ sitting job, CT: computed tomography, PET: positron emission tomography

### Measurement of BP and HR

SBP, DBP, and HR were measured using a wristwatch-type WD (itDEAL Smart Watch W11; itDEAL, China). The device can measure SBP and DBP every 10 minutes and HR every 30 minutes using optical measurement technology.

To stabilize the measurements, participants wore the device on the left wrist for approximately one week during work time. After this period, all participants attended an interview in which they were asked about their medical history and medications, as well as their job, workplace (division), and their years of experience overall and in that division. Participants selected their job and workplace from a list, and recorded these details using a provided Excel spreadsheet for each daily measurement period.

With the participant seated and with both arms resting on the table at a similar level to the heart, we recorded SBP, DBP, and HR three times at the right upper arm of each participant using a conventional oscillometric-type sphygmomanometer and pulse monitor measurement device approved in Japan for clinical use (UA-772B; A&D Co. Ltd., Tokyo, Japan). Baseline WD measurements of SBP, DBP, and HR were recorded simultaneously three times at the left wrist, at 17:00. Measurements of SBP, DBP, and HR were then begun on workdays between 7:00 and 18:00, and were recorded during any consecutive 8-week period between May and October 2021.

### Data migration

Measurement data from the WDs were sent to smartphones (Geanee ADP-503G; JENESIS Co. Ltd., Tokyo, Japan) onto which the H Band 2.0 recording application was downloaded. Each WD was linked to an individual smartphone via Bluetooth. The smartphones were used only to obtain data from WD. They had no call function and were restricted from connecting to any network except Bluetooth. All measurement data recorded by the application were sent to an Excel spreadsheet for further analysis. The measurement data were then collated with those of hours of work, day of the week, job designation, and workplace recorded by the participants.

### Data analysis

Measurement data obtained between 8:00 and 17:00 were used in the analysis to ensure consistency of the data. No additional data were introduced to adjust for defects in any measurement results. In addition to SBP, DBP, and HR, we also calculated pulse pressure (PP), defined as the difference between SBP and DBP. The SBP, DBP, PP, and HR values are presented as the mean (± SD), maximum, minimum, and coefficient of variation [CV(%)], each of which was normalized as the ratio to baseline (measurement value divided by the mean baseline value). To evaluate the influence of body position on the measurement data, each job was categorized as a “standing job” or “sitting job”, as shown in Table 1.

Unpaired t-test was used to compare the WD measurement data at 17:00 with the baseline values, and to compare measurement data between sitting and standing jobs. Pearson correlation coefficient (r) was used to evaluate the relationship between the basic measurements obtained by WD and those by medical sphygmomanometer. Bland-Altman plot was used to evaluate the degree of agreement between the basic measurements obtained by WD and those by medical sphygmomanometer.

## Results

### Comparison of baseline and work time WD measurements

**Fig 1:**
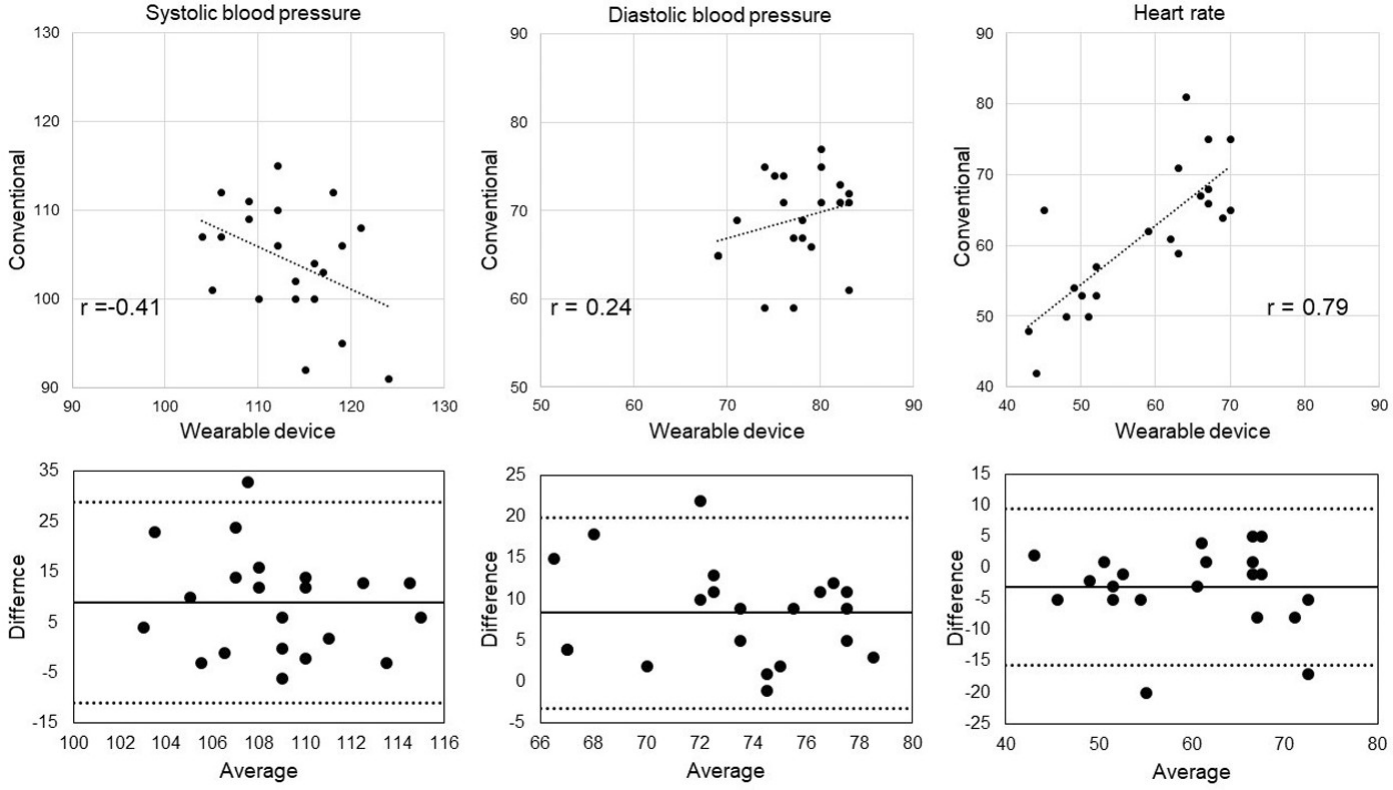
Correlation and degree of agreement between measurements obtained by the oscillometric type sphygmomanometer (conventional) and WD. upper low: Pearson correlation coefficient analysis (r and black solid line), lower low: Bland-Altman plot analysis The mean difference is represented by the black solid line with the 95% limits of agreement represented by the dashed lines.

**Fig 2:**
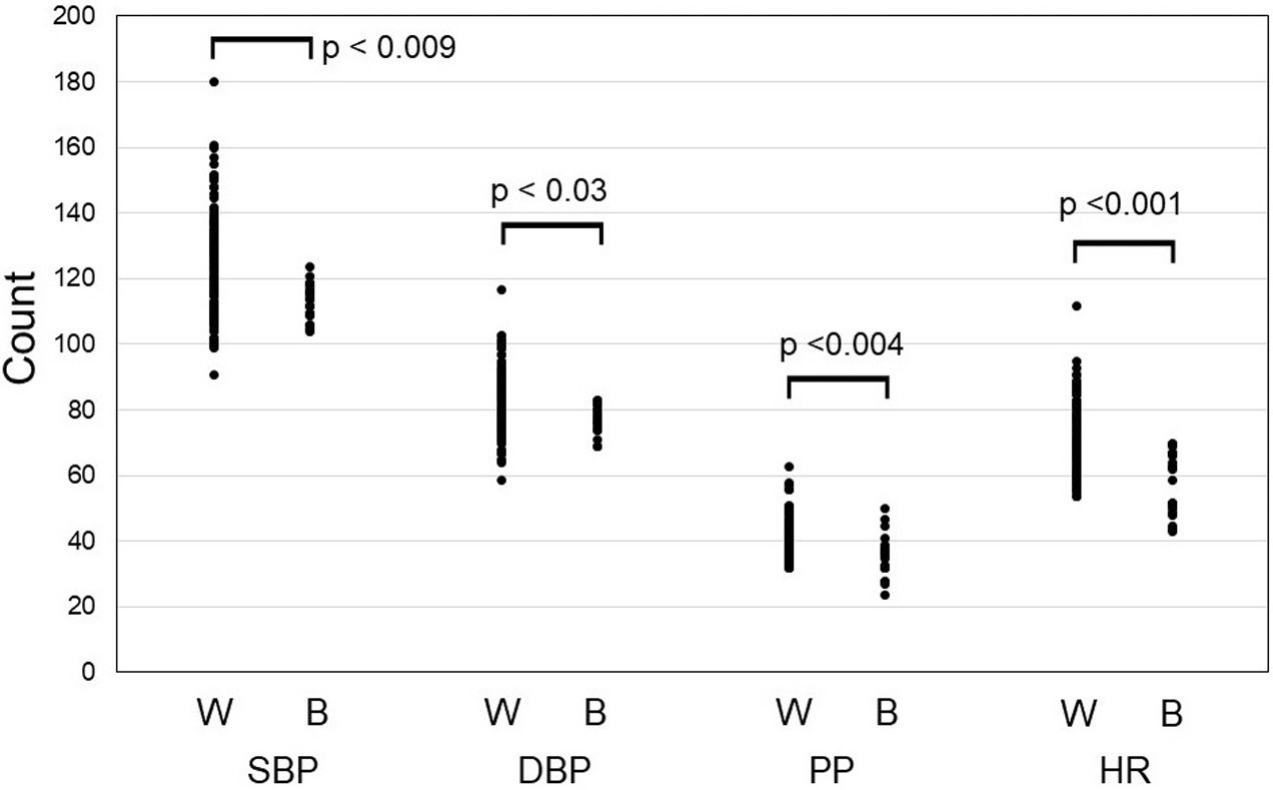
Measurements obtained at work time by WD and baseline measurements.

The difference between the oscillometric type sphygmomanometer and WD were 9 ± 10 mmHg in SBP, 8 ± 6 mmHg in DBP and -3 ± 6 in HR. Correlation between measurements obtained by the oscillometric type sphygmomanometer and WD was r = –0.41 for SBP, r = 0.24 for DBP, and r = 0.79 for HR (Fig 1: upper low). The agreement of measurement results between the oscillometric type sphygmomanometer and WD is graphically depicted with a Bland-Altman in Fig 1 (lower low). Measurements obtained at 17:00 during work time by WD were significantly higher than the baseline measurements (measured at 17:00) for SBP, DBP, and HR (Fig 2).

### Measurement results in individuals

The measurements obtained in each participant are shown in Tables 2, and 3 shows the ratio to baseline of the mean, max/min, and CV(%) values for each metric, for each participant. The ratio to baseline ranged from 1.02 to 1.26 for SBP and from 1.07 to 1.30 for DBP, and SBP was >130 mmHg in two of the seven participants. The ratio to baseline of CV(%) for SBP and DBP was approximately 10% over the total observation term and per day. The ratio to baseline for HR ranged from 0.95 to 1.29, and mean HR was >70 bpm in four of the seven participants. CV(%) of HR was almost the same as that for SBP, DBP, and PP. Among the participants, SBP and DBP values showed similar trends, and thus PP showed little variation. The trends in SBP and DBP for individuals were not consistent with the trends in HR.

**Table 2:** Measurement result of subjects.

Index		Participants						
		A	B	C	D	E	F	G
SBP	Average ± SD	131 ± 14	110 ± 2	126 ± 14	125 ± 13	132 ± 11	117 ± 15	123 ± 15
	Max/Min	181/87	119/105	182/94	170/100	201/100	183/91	177/95
	CV (%)	10.8	1.6	11.3	10.1	8.4	12.8	12.1
	CV(%) per day	10.9	1.5	11.2	9.8	8.1	12.5	12.0
DBP	Average ± SD	87 ± 9	76 ± 1	84 ± 9	83 ± 8	88 ± 7	78 ± 10	82 ± 10
	Max/Min	118/56	81/73	119/61	110/67	132/67	120/59	115/61
	CV (%)	10.2	1.2	11.0	9.8	8.0	13.1	11.9
	CV(%) per day	10.3	1.2	10.9	9.5	7.7	12.8	11.7
PP	Average ± SD	44 ± 5	34 ± 1	42 ± 5	41 ± 5	44 ± 4	39 ± 5	41 ± 5
	Max/Min	63/31	38/31	101/33	60/33	69/33	63/32	62/33
	CV (%)	12.4	2.9	12.4	11.0	9.4	12.6	12.9
	CV(%) per day	12.5	2.8	12.1	10.7	9.0	12.3	12.7
HR	Average ± SD	72 ± 8	80 ± 7	67 ± 9	71 ± 7	71 ± 10	66 ± 9	68 ± 8
	Max/Min	95/58	112/65	103/55	101/58	105/57	104/49	105/56
	CV (%)	10.8	9.1	14.0	10.2	14.4	13.8	11.3
	CV(%) per day	8.0	7.6	10.3	9.1	10.1	11.0	10.0

SBP: systolic blood pressure, DBP: diastolic blood pressure, PP: pulse pressure, HR: heart rate, CV: coefficient of variation, CV(%) per day: average of CV in each day.

**Table 3:** Ratio of measurement result compared to the baseline.

Index		Participants						
		A	B	C	D	E	F	G
SBP	Average ± SD	1.19 ± 0.13	1.02 ± 0.02	1.16 ± 0.13	1.18 ± 0.12	1.19 ± 0.10	1.26 ± 0.16	1.21 ± 0.15
	Max/Min	1.65 / 0.79	1.11 / 0.98	1.67 / 0.87	1.61 / 0.95	1.80 / 0.90	1.97 / 0.98	1.74 / 0.93
	CV(%)	10.8	1.6	11.3	10.1	8.4	12.8	12.1
DBP	Average ± SD	1.19 ± 0.12	1.07 ± 0.01	1.16 ± 0.13	1.18 ± 0.12	1.18 ± 0.09	1.30 ± 0.17	1.22 ± 0.14
	Max/Min	1.61 / 0.76	1.13 / 1.01	1.64 / 0.84	1.56 / 0.95	1.77 / 0.90	2.01 / 0.99	1.71 / 0.91
	CV(%)	10.2	1.2	11.0	9.8	8.0	13.1	11.9
HR	Average ± SD	1.02 ± 0.11	1.17 ± 0.11	0.95 ± 0.14	1.17 ± 0.12	1.09 ± 0.16	1.29 ± 0.18	1.25 ± 0.14
	Max/Min	1.34 / 0.82	1.63 / 0.95	2.30 / 0.77	1.66 / 0.96	1.62 / 0.88	2.01 / 0.95	1.93 / 1.01
	CV(%)	10.8	9.1	14.0	10.2	14.4	13.8	11.3

SBP: systolic blood pressure, DBP: diastolic blood pressure, HR: heart rate, CV: coefficient of variation

### Measurement deficit rates

Table 4 shows the measurement deficit rates, which ranged from 17.6% to 37.7% for BP according to work description and from 16.2% to 44.0% by work location. There was minor deficit of measurement for HR. There was no deficit of measurement in specific individuals. The deficit rate was 24.1% for sitting jobs and 27.0% for standing jobs.

**Table 4:** Deficit rates of measurement data by job and workplace.

Group	Item	Deficit rate of BP (%)	Deficit rate of HR (%)
Job	Preparation of radiopharmaceuticals	37.7	2.2
	Equipment check	33.9	0
	Registration	28.6	0
	Irradiation	28.1	0
	Rest	27.1	1.5
	CT scan for therapeutic Planning	27.0	0
	Scanning	26.3	0
	Dose verification	25.2	0
	Equipment accuracy control	24.4	0
	Radiotherapy planning	22.0	0
	Dealing with patients	20.2	0
	Research	21.6	0
	Data analysis	18.8	0
	Conference	17.6	0
Workplace	Lunchroom	44.0	5.1
	Preparation room for radiopharmaceutical	31.3	2.4
	CT examination room	26.5	0
	Linac room	26.5	0
	PET examination room	23.9	0
	Scintigraphy examination room	23.7	0
	Office	23.1	0
	Treatment planning room	16.2	0

BP: blood pressure, HR: heart rate, CT: computed tomography, PET: positron emission tomography

### Variation in BP and HR by time and day of week

**Fig 3:**
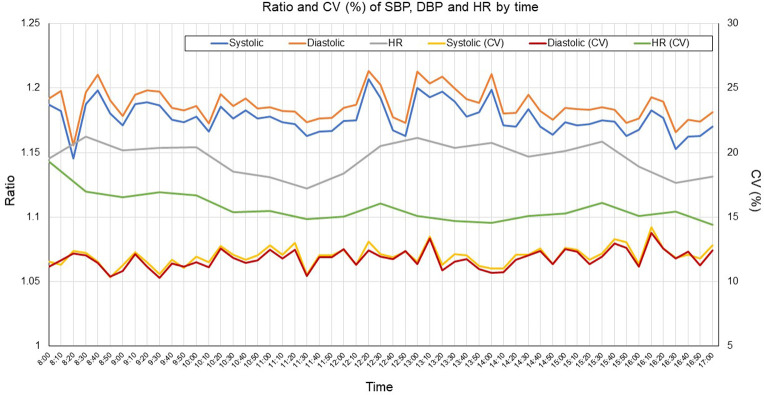
Ratio and CV (%) of SBP, DBP and HR by time.

**Fig 4:**
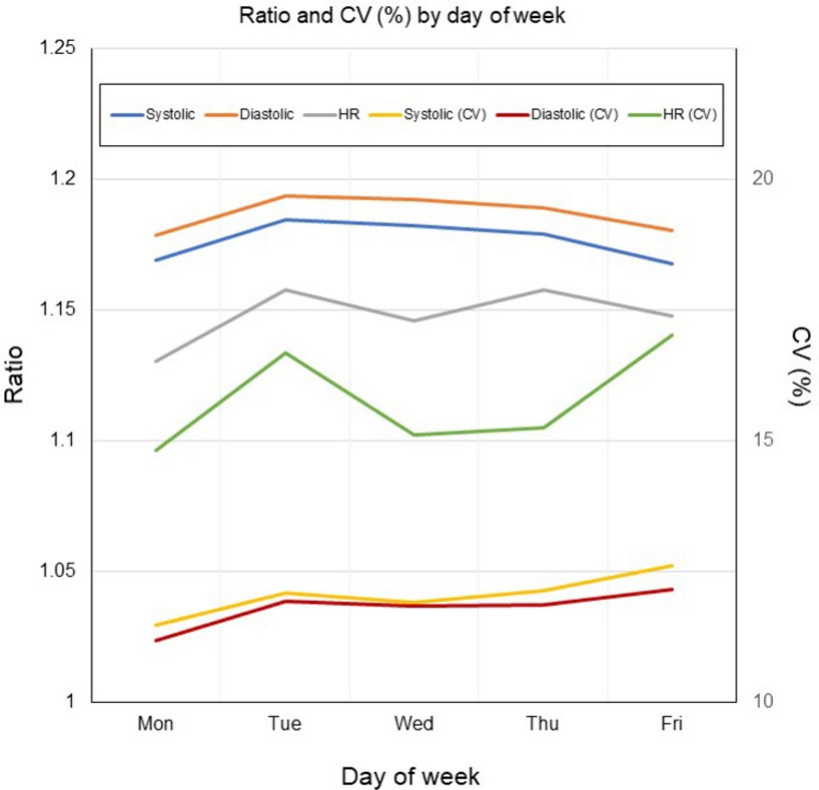
Ratio and CV (%) of SBP, DBP and HR by day of week.

For BP, the ratio of the mean measurements to baseline was almost 1.15 to 1.2 but was >1.2 at the time of starting work, middle of lunch time, after lunchtime, and at 14:00; and the ratio to baseline for CV(%) showed a similar trend as the ratio to baseline of the mean values. For HR, the ratio to baseline decreased in the late morning and the CV(%) values were generally stable at all times (Fig 3). The ratios to baseline of SBP and DBP were generally stable, whereas that of HR showed variation according to the day of the week (Fig 4).

### Variation in BP and HR according to job and workplace

**Fig 5:**
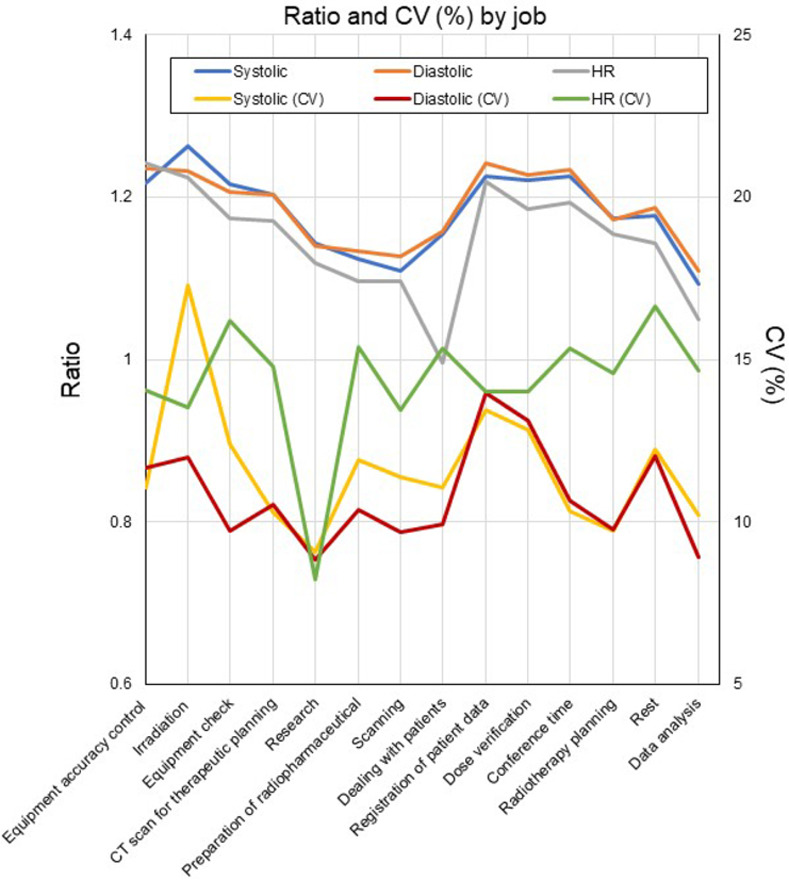
Ratio and CV (%) of SBP, DBP and HR by job.

The ratio to baseline for SBP, DBP, and HR was >1.2 for irradiation, equipment accuracy control, registration of patient data, dose verification, and conference time; and was <1.2 for scanning, research, preparation of radiopharmaceuticals, dealing with patients, and data analysis (Fig 5).

**Fig 6:**
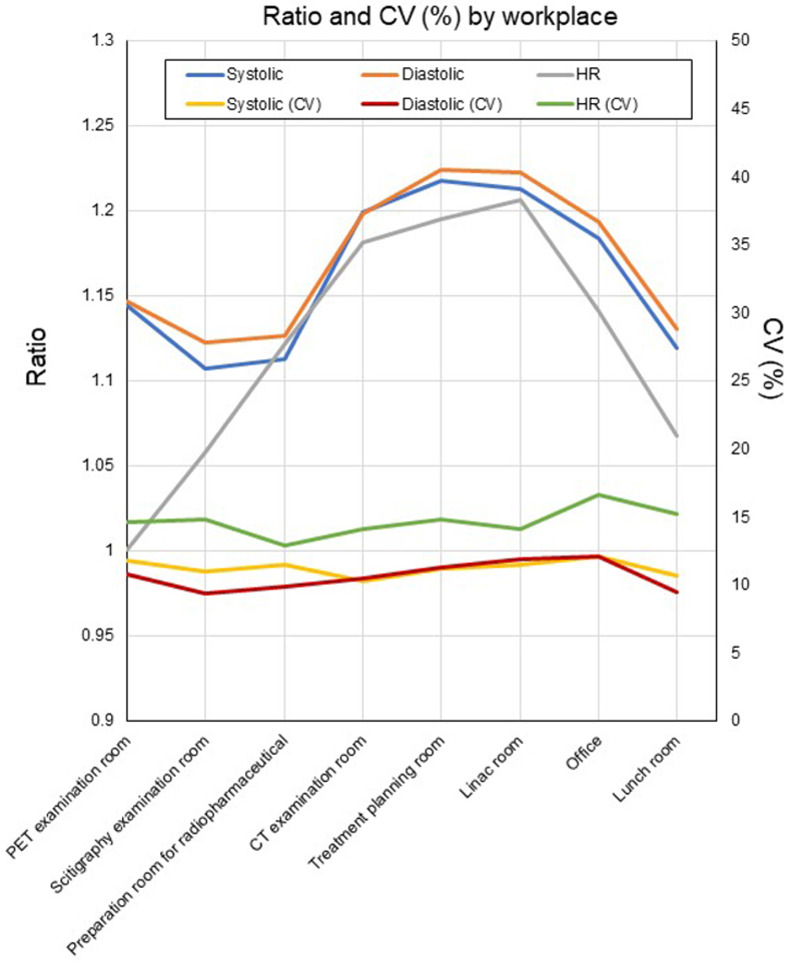
Ratio and CV (%) of SBP, DBP and HR by workplace.

The ratio to baseline for SBP, DBP and HR was significantly higher for jobs in the standing position than the sitting position (SBP: standing position 1.23±0.19, sitting position 1.15±0.13, p<0.001, DBP: standing position 1.22±0.14, sitting position 1.17±0.13, p<0.001, HR: standing position 1.18±0.18, sitting position 1.14±0.18, p<0.001). CV(%) was higher in the standing position than the sitting position for SBP, but was similar between positions for DBP and HR (SBP: standing position 15.5, sitting position 11.7, DBP: standing position 11.8, sitting position 11.5, HR: standing position 15.6, sitting position 15.7). The ratio to baseline for SBP, DBP, and HR was higher for the locations of CT examination room, treatment planning room, linac room, and the office, and lower for the lunchroom. The CV(%) values of these metrics were similar for all workplaces (Fig 6). The detailed measurement data according to the day of the week, job, and workplace are provided in S1–S3 Tables, respectively.

## Discussion

In this preliminary study, we obtained BP and HR of young radiological technologists measured using a WD, and evaluated variation in the values among individuals, and according to the day of the week, job, and workplace. Measurements obtained during work time by WD were significantly higher than those at baseline for SBP, DBP, and HR. The ratio of mean to baseline measurements ranged from 1.02 to 1.26 for SBP, from 1.07 to 1.30 for DBP, and from 0.95 to 1.29 for HR. The ratio of mean BP was >1.2 at the time of starting work, middle of lunch time, after lunch, and at 14:00. The ratio to baseline for SBP, DBP, and HR was >1.2 for irradiation, equipment accuracy control, registration of patient data, dose verification, and conference time. These values were also higher for the locations of CT examination room, treatment planning room, linac room and the office. CV(%) values of these metrics were generally stable for all workplaces. Measurement of these metrics by WD may be a useful method for evaluating an individual’s unconscious workload.

The WD could record BP and HR measurements periodically with minimal discomfort and without interfering with work. The collection of a large amount of data by WD might enable the discovery of characteristics that lead to present or future pathological conditions. In addition, the simultaneous monitoring of environmental conditions and WD measurement enables detection of values indicative of abnormal condition. Therefore, the shift to the digital approach of monitoring BP and HR is expected to make a significant contribution to the field of preventive medicine [21, 22].

Photoplethysmography (PPG) is a noninvasive optical technique using infrared light and photodiodes to visualize the pressure pulse waves (PWs) in blood vessels by measuring the volumetric changes of pulsating blood and thus the expansion and contraction of the vessels [23, 24]. With the advancement of digital sensors, signal processing, machine-learning algorithms, and improved physiologic models, pulse waveform analysis to assess BP has become feasible [25–29].

However, sensing, biological, and cardiovascular factors can affect PPG recordings. Tissue modifications generated by voluntary movements can create alterations of inner tissues, which leads to modification of receiving light resulting in generating a different signal. Therefore, BP measurement results with WD may be impacted by stationary or not at the moment of BP measurement [30].

It is known that commercially available mobile health devices that record measurements have limited reliability [31], and that a large proportion of devices marketed for BP monitoring have not been sufficiently validated [32]. Our results showed that baseline BP did not correlate highly enough with those obtained by the medical sphygmomanometer. Radial and brachial SBP measurements differ, with average values reported as 5.5 mm Hg higher for radial SBP than brachial SBP [33]. In our study, mean (±SD) SBP (113±6) and DBP (77±4) measured at the left wrist by WD tended to be higher than those measured at the upper arm by a medical sphygmomanometer (104±7 and 69±5, respectively), but the correlation between them was low to modest. In addition, there was a deficit of measurement for SBP and DBP. Therefore, greater accuracy is required in BP measurements obtained with a WD. In contrast, there was good correlation between the device types for HR, with almost no measurement deficit.

Mean SBP and DBP measurements by WD were higher than baseline in all participants and showed difference in CV(%) of approximately 10%. Mean SBP was >130 mmHg in two participants, which is higher than normal. Comparison of measurements obtained at 17:00 revealed that mean BP, PP and HR values were significantly higher than at baseline, which suggests an influence of work-related factors.

Because the baseline measurements showed individual variation in BP and HR, we used the ratio to baseline (measurement value divided by the mean baseline value) to normalize the participants’ data. The measurements for SBP obtained at work were 1.02–1.26 times higher than the baseline values. Job strain appeared to be a trigger for elevation of SBP.

In general, BP tends to be higher in the morning and lower at night, and there is interindividual variation in daytime BP in response to stress [2]. Our study showed that ratio to baseline values for BP were highest at the start of work, lunchtime, start of afternoon work time, and early afternoon. The ratio to baseline values for HR were also highest at the start of work and in the afternoon. This finding suggests that starting work was one of the factors that increased the ratio to baseline values of BP and HR. Day of the week had little influence on the ratio of mean BP to baseline, and a slight influence on HR. Although the season of the year might have an effect on BP, our measurements were all conducted in the summer season and therefore we consider that season would not have affected our data.

In terms of job performed, the ratio to baseline for BP was higher for irradiation, registration of patient data, dose verification and conference time, and lower for research, preparation of radiopharmaceuticals, scanning, and dealing with patients, which was possibly due to the radiological technologist’s body position: BP was higher in jobs performed in the standing position than the sitting position, whereas there was little difference in HR between positions. BP is rarely measured in the standing position; however, SBP has been reported to decrease by approximately 8 mmHg when an individual changes their position from lying to sitting or standing [34, 35]. Another study found no difference in SBP or DBP according to body position in participants in their 20s and 30s, but a difference was found in those in an older age group [36]. Thomas et al. reported a more frequent incidence of hypertension within 8 years in young adults who showed an increase in SBP after changing to the standing position [37]. Based on these results, an increase in SBP while working in the standing position may be a notable finding for monitoring the state of health. In contrast, it has been generally recommended that prolonged sitting during deskwork should be reduced; therefore, investigation of the benefits of a balance of sitting and standing work may be task for the future [38]. The effects of HR monitoring remain unclear because interrupting prolonged sitting leads to a non-significant increase in HR [39]. Other possible explanations for the variation of B is that BP might be influenced by the jobs that require quick and precise decision-making. In contrast, the body can recover BP during jobs that take longer time. Rest time has a certain effect on stabilizing blood pressure, because the ratio to baseline value for rest time was in the middle of the range among the various jobs. However, it should be understood that moving from rest to working is the point when BP will change largely.

This study has several limitations, which include the small number of participants, and that continuous data collection with large population is still required. The WD used in this study was a commercially available product, and a greater degree of quality control is required regarding accuracy. Our results might have been affected by how the WD was worn, and factors such as tightness on the wrist, slight changes in the position of the WD while working, and skin dryness require investigation in future studies.

## Supporting information

S1 Table Measurement result by day of week. (DOCX)

S2 Table Measurement result by job. (DOCX)

S3 Table Measurement result by workplace. (DOCX)
