# Supplementary material for: Variation in blood pressure and heart rate of radiological technologists in worktime tracked by a wearable device: A preliminary study
Source: PLoS One. 2022 Nov 17;17(11):e0276483. doi: 10.1371/journal.pone.0276483 (PMC9671413; doi:10.1371/journal.pone.0276483)
Supplement: S1 Table — (DOCX) [file pone.0276483.s001.docx]

Supplement Table 1. Measurement result by day of week

| Index |  | Monday | Tuesday | Wednesday | Thursday | Friday |
| --- | --- | --- | --- | --- | --- | --- |
| SBP | Average ± SD | 122 ± 14 | 124 ± 15 | 123 ± 14 | 123 ± 15 | 122 ± 15 |
|  | Max/Min | 181/87 | 181/91 | 180/93 | 201/91 | 183/94 |
|  | CV (%) | 11.7 | 11.9 | 11.7 | 12.0 | 12.0 |
|  | CV(%) per person | 9.4 | 9.7 | 9.3 | 9.7 | 9.5 |
| DBP | Average ± SD | 82 ± 9 | 83 ± 9 | 82 ± 9 | 82 ± 9 | 82 ± 9 |
|  | Max/Min | 118/56 | 118/59 | 117/60 | 132/59 | 120/61 |
|  | CV (%) | 11.2 | 11.4 | 11.2 | 11.3 | 11.0 |
|  | CV(%) per person | 9.3 | 9.5 | 9.0 | 9.3 | 9.1 |
| PP | Average ± SD | 41 ± 6 | 41 ± 6 | 41 ± 5 | 41 ± 6 | 40 ± 6 |
|  | Max/Min | 63/31 | 63/31 | 63/31 | 101/31 | 63/31 |
|  | CV (%) | 13.5 | 13.6 | 13.1 | 14.4 | 14.6 |
|  | CV(%) per person | 10.2 | 10.5 | 10.2 | 10.9 | 10.6 |
| HR | Average ± SD | 70 ± 10 | 71 ± 10 | 71 ± 10 | 71 ± 9 | 71 ± 10 |
|  | Max/Min | 101/52 | 105/49 | 112/53 | 109/49 | 104/52 |
|  | CV (%) | 14.2 | 14.4 | 13.7 | 13.0 | 13.5 |
|  | CV(%) per person | 11.8 | 13.0 | 11.4 | 11.1 | 11.2 |

SBP: systolic blood pressure, DBP: diastolic blood pressure, PP: pulse pressure, HR: heart rate, CV: coefficient of variation, CV(%) per person: average of CV in each person.
