# Supplementary material for: Variation in blood pressure and heart rate of radiological technologists in worktime tracked by a wearable device: A preliminary study
Source: PLoS One. 2022 Nov 17;17(11):e0276483. doi: 10.1371/journal.pone.0276483 (PMC9671413; doi:10.1371/journal.pone.0276483)
Supplement: S2 Table — (DOCX) [file pone.0276483.s002.docx]

Supplement Table 2 Measurement result by job

| Index |  | Dose verification | Equipment accuracy control | Data analysis | Equipment check | Dealing with patients | Conference  time | Scanning | Rest | Preparation of  radiopharmaceuticals | Research | Radiotherapy  planning | CT scan for therapeutic  planning | Irradiation | Registration of patient data |
| --- | --- | --- | --- | --- | --- | --- | --- | --- | --- | --- | --- | --- | --- | --- | --- |
| SBP | Average ± SD | 126 ± 15 | 122 ± 14 | 118 ± 13 | 124 ± 15 | 125 ± 14 | 127 ± 14 | 120 ± 14 | 123 ± 15 | 122 ± 15 | 121 ± 11 | 127 ± 13 | 128 ± 14 | 123 ± 15 | 122 ± 16 |
|  | Max/Min | 180/99 | 174/91 | 181/100 | 160/100 | 171/100 | 165/65 | 182/87 | 171/94 | 174/105 | 162/101 | 180/96 | 163/95 | 201/91 | 180/93 |
|  | CV (%) | 11.8 | 11.5 | 10.8 | 11.9 | 11.4 | 11.0 | 12.0 | 11.8 | 12.4 | 9.2 | 9.9 | 10.6 | 12.2 | 13.4 |
|  | CV(%) per person | 11.4 | 10.1 | 7.7 | 9.5 | 7.3 | 10.0 | 7.9 | 9.6 | 9.1 | 6.2 | 11.3 | 10.6 | 10.9 | 11.7 |
| DBP | Average ± SD | 84 ± 10 | 81 ± 9 | 80 ± 8 | 82 ± 10 | 84 ± 9 | 85 ± 9 | 81 ± 8 | 82 ± 9 | 82 ± 9 | 81 ± 7 | 85 ± 8 | 85 ± 9 | 81 ± 10 | 81 ± 11 |
|  | Max/Min | 117/64 | 113/59 | 118/66 | 103/65 | 111/65 | 106/61 | 119/56 | 111/61 | 113/70 | 104/67 | 117/62 | 105/61 | 132/59 | 117/60 |
|  | CV (%) | 11.4 | 11.6 | 9.4 | 11.8 | 10.3 | 10.8 | 10.2 | 11.2 | 10.8 | 9.0 | 9.7 | 10.4 | 12.2 | 13.4 |
|  | CV(%) per person | 11.1 | 10.1 | 7.4 | 9.4 | 6.9 | 9.7 | 7.5 | 9.3 | 8.3 | 6.2 | 11.1 | 10.7 | 10.7 | 11.6 |
| PP | Average ± SD | 42 ± 5 | 41 ± 5 | 38 ± 6 | 42 ± 5 | 41 ± 6 | 43 ± 5 | 39 ± 6 | 41 ± 6 | 40 ± 6 | 40 ± 4 | 43 ± 5 | 43 ± 5 | 41 ± 5 | 41 ± 6 |
|  | Max/Min | 63/33 | 61/32 | 63/31 | 57/33 | 60/32 | 59/33 | 63/31 | 60/31 | 61/32 | 58/34 | 63/33 | 58/33 | 69/32 | 63/33 |
|  | CV (%) | 12.8 | 11.5 | 14.5 | 12.4 | 14.3 | 11.8 | 16.2 | 13.8 | 16.2 | 9.8 | 10.6 | 11.3 | 12.5 | 13.7 |
|  | CV(%) per person | 12.5 | 10.3 | 8.5 | 10.0 | 8.5 | 10.9 | 9.1 | 10.6 | 10.8 | 6.4 | 11.9 | 10.8 | 11.5 | 12.3 |
| HR | Average ± SD | 68 ± 9 | 69 ± 9 | 73 ± 10 | 68 ± 9 | 70 ± 10 | 69 ± 10 | 76 ± 10 | 71± 10 | 76 ± 11 | 68 ± 5 | 72 ± 10 | 71 ± 9 | 68 ± 8 | 67 ± 9 |
|  | Max/Min | 91/52 | 93/52 | 98/55 | 100/53 | 109/56 | 104/54 | 103/56 | 105/52 | 98/58 | 78/58 | 95/56 | 103/55 | 105/49 | 92/51 |
|  | CV (%) | 12.8 | 13.0 | 14.0 | 12.7 | 14.7 | 14.1 | 12.5 | 13.9 | 14.5 | 8.0 | 13.7 | 13.2 | 12.3 | 12.8 |
|  | CV(%) per person | 12.4 | 11.7 | 10.9 | 12.2 | 11.8 | 13.1 | 10.6 | 13.0 | 12.8 | 8.1 | 13.3 | 11.8 | 12.2 | 11.7 |

SBP: systolic blood pressure, DBP: diastolic blood pressure, PP: pulse pressure, HR: heart rate, CV: coefficient of variation, CV(%) per person: average of CV in each person, CT: computed tomography.
