# Supplementary material for: Variation in blood pressure and heart rate of radiological technologists in worktime tracked by a wearable device: A preliminary study
Source: PLoS One. 2022 Nov 17;17(11):e0276483. doi: 10.1371/journal.pone.0276483 (PMC9671413; doi:10.1371/journal.pone.0276483)
Supplement: S3 Table — (DOCX) [file pone.0276483.s003.docx]

Supplement Table 3. Measurement result by workplace

| Index |  | PET examination room | Scintigraphy examination room | Preparation room for　 Radiopharmaceutical | CT examination  room | Treatment planning  room | Linac room | Office | Lunchroom |
| --- | --- | --- | --- | --- | --- | --- | --- | --- | --- |
| SBP | Average ± SD | 125 ± 15 | 120 ± 14 | 121 ± 15 | 127 ± 14 | 126 ± 15 | 123 ± 15 | 125 ± 15 | 120 ± 14 |
|  | Max/Min | 170/98 | 182/87 | 174/105 | 163/95 | 180/95 | 201/91 | 180/96 | 165/94 |
|  | CV (%) | 12.1 | 11.5 | 12.1 | 10.6 | 11.8 | 12.0 | 11.7 | 11.4 |
|  | CV(%) per person | 8.2 | 7.6 | 8.3 | 10.7 | 10.5 | 10.8 | 12.5 | 8.7 |
| DBP | Average ± SD | 83 ± 9 | 81 ± 8 | 82 ± 8 | 85 ± 9 | 84 ± 10 | 82 ± 10 | 83 ± 10 | 81 ± 8 |
|  | Max/Min | 110/63 | 119/56 | 113/70 | 105/61 | 117/61 | 132/59 | 117/62 | 107/61 |
|  | CV (%) | 11.0 | 9.9 | 10.3 | 10.5 | 11.5 | 11.9 | 11.6 | 10.1 |
|  | CV(%) per person | 7.8 | 7.3 | 7.7 | 10.8 | 10.2 | 10.7 | 12.5 | 8.2 |
| PP | Average ± SD | 41 ± 6 | 39 ± 6 | 39 ± 6 | 43 ± 5 | 42 ± 5 | 42 ± 5 | 42 ± 5 | 39 ± 6 |
|  | Max/Min | 60/32 | 101/31 | 61/32 | 58/33 | 63/33 | 69/32 | 63/33 | 60/31 |
|  | CV (%) | 14.9 | 15.8 | 16.3 | 11.3 | 12.6 | 12.3 | 12.5 | 15.1 |
|  | CV(%) per person | 9.5 | 9.0 | 10.0 | 10.8 | 11.4 | 11.4 | 12.9 | 10.0 |
| HR | Average ± SD | 70 ± 10 | 74 ± 10 | 78 ± 10 | 72 ± 9 | 69 ± 9 | 69 ± 9 | 68 ± 8 | 75 ± 12 |
|  | Max/Min | 99/55 | 112/55 | 98/58 | 103/55 | 104 | 105/49 | 104/52 | 105/57 |
|  | CV (%) | 13.7 | 13.9 | 12.2 | 12.7 | 55 | 12.9 | 12.3 | 14.1 |
|  | CV(%) per person | 10.2 | 11.4 | 9.8 | 11.5 | 13.3 | 12.3 | 12.1 | 12.8 |

SBP: systolic blood pressure, DBP: diastolic blood pressure, PP: pulse pressure, HR: heart rate, CV: coefficient of variation, CV(%) per person: average of CV in each person, CT: computed tomography, PET: positron emission tomography
